# Dynamics of amygdala connectivity in bipolar disorders: a longitudinal study across mood states

**DOI:** 10.1038/s41386-021-01038-x

**Published:** 2021-06-07

**Authors:** Gwladys Rey, Thomas A. W. Bolton, Julian Gaviria, Camille Piguet, Maria Giulia Preti, Sophie Favre, Jean-Michel Aubry, Dimitri Van De Ville, Patrik Vuilleumier

**Affiliations:** 1 Laboratory for Behavioral Neurology and Imaging of Cognition, Department of Fundamental Neurosciences, University of Geneva, Geneva, Switzerland; 2 Institute of Bioengineering, École Polytechnique Fédérale de Lausanne (EPFL), Lausanne, Switzerland; 3 Department of Psychiatry, University Hospitals of Geneva, Geneva, Switzerland; 4 Department of Radiology and Medical Informatics, University of Geneva, Geneva, Switzerland

**Keywords:** Bipolar disorder, Diagnostic markers

## Abstract

Alterations in activity and connectivity of brain circuits implicated in emotion processing and emotion regulation have been observed during resting-state for different clinical phases of bipolar disorders (BD), but longitudinal investigations across different mood states in the same patients are still rare. Furthermore, measuring dynamics of functional connectivity patterns offers a powerful method to explore changes in the brain’s intrinsic functional organization across mood states. We used a novel co-activation pattern (CAP) analysis to explore the dynamics of amygdala connectivity at rest in a cohort of 20 BD patients prospectively followed-up and scanned across distinct mood states: euthymia (20 patients; 39 sessions), depression (12 patients; 18 sessions), or mania/hypomania (14 patients; 18 sessions). We compared them to 41 healthy controls scanned once or twice (55 sessions). We characterized temporal aspects of dynamic fluctuations in amygdala connectivity over the whole brain as a function of current mood. We identified six distinct networks describing amygdala connectivity, among which an interoceptive-sensorimotor CAP exhibited more frequent occurrences during hypomania compared to other mood states, and predicted more severe symptoms of irritability and motor agitation. In contrast, a default-mode CAP exhibited more frequent occurrences during depression compared to other mood states and compared to controls, with a positive association with depression severity. Our results reveal distinctive interactions between amygdala and distributed brain networks in different mood states, and foster research on interoception and default-mode systems especially during the manic and depressive phase, respectively. Our study also demonstrates the benefits of assessing brain dynamics in BD.

## Introduction

To achieve good monitoring of bipolar disorder (BD) patients’ state and prognosis, psychiatric research needs to better characterize the neural processes that distinguish different clinical phases of the disease. Resting-state brain imaging studies of BD, initially limited to Independent Component Analysis (ICA) and seed-based functional connectivity (FC), and later enriched by graph theory methods, have contributed to the demonstration of widespread FC disruptions [1–3]. This literature incriminates different brain areas and networks such as the default-mode network, limbic and reward circuits, and more recently sensorimotor networks [4–7]. In particular, the amygdala, already known for its central role in models of BD [8, 9], undergoes abnormal interactions with the medial prefrontal cortex, posterior cingulate cortex, and both ventro- and dorso-lateral prefrontal cortices at rest [10–12]. However, these findings still lack consistency and reproducibility, partly because different studies recruited patients in different clinical phases and did not perform systematic comparisons between mood states, thus hindering the distinction between trait effects (diagnosis-specific) and state effects (mood-specific). Moreover, although a few investigations segregated different states, they compared different patients [2, 7, 13] rather than the same patients across different phases.

Therefore, the first aim of our study was to investigate FC at rest in BD patients by obtaining repetitive/periodic scans within the *same* individuals at different phases of the disease. This approach has so far only been adopted in a couple of task-based studies [e.g., 14, 15], and in one resting-state study including ten patients scanned in both mania and euthymia [16]. However, to our knowledge, the need to perform longitudinal comparisons in the same patients across all possible states (from low through normal to high mood) is still unmet.

A second aim of our study was to adopt a novel dynamic approach by considering the “non-stationarity” of the resting brain. Since spontaneous brain FC may dynamically change over time [17, 18], a number of methodological developments have tried to track these temporal fluctuations. However, most studies employed a sliding-window approach, i.e., using successive averages of connectivity values derived from between-area correlations [19]. Previous applications of the latter method to psychotic disorders showed that brain connectivity dynamics provides more discriminant information than static measures computed over a whole scanning run, in the context of automatic classification of mental disorders [20, 21]. However, the sliding-window correlation approach implies a computation of FC over discrete, consecutive segments of the data, carrying certain limitations especially with regard to the effective temporal resolution of the analysis and the need for parameter selection [see review in 19].

In the present study, we propose to use an alternative method, based on “co-activation patterns” or CAPs [22], which departs from the sliding-window approach by assuming that the most informative activity of the brain can be captured by a limited amount of data [23]. Indeed, work by Tagliazucchi et al. demonstrated that most of the time, the brain fluctuates near the ‘critical point’ (equilibrium), and that a point process analysis (PPA), selecting only a few relevant time points (i.e., when the signal exceeds a certain threshold), can produce findings similar to those obtained when the entire set of time points is considered. In the CAP method, the PPA is applied to a single seed, by retaining solely the brain volumes (with un-thresholded activity) at time points when the seed is particularly active [22]. A temporal clustering is then applied to the selected frames. Spatial averaging of the frames grouped together yields different CAPs; i.e., sets of brain areas that intermittently co-activate or co-deactivate with the seed.

Here, we applied the CAP method using the toolbox *TbCAPs* [24] to explore the dynamics of amygdala connectivity in our longitudinally followed bipolar disorder patients. The sensitivity and potential specificity of the amygdala to affective episodes in BD [2, 10, 25], and more generally its major involvement in emotion appraisal and emotion regulation processes [26], motivated our choice of this structure as a seed in order to probe for differential connectivity patterns across mood fluctuations. We expected that the dynamics of amygdala connectivity would not only unveil its interactions with multiple brain networks—but also reflect the clinical state of the patients, especially when comparing affective episodes with normalized mood.

## Material and methods

### Participants

The study protocol and the informed consent procedure received approval from the ethics committee of the Geneva University Hospitals. Table 1 provides demographic and clinical description of the patients and controls samples.

**Table 1:** Demographic and clinical description of the participants.

	Bipolar disorders patients (BP) *N* = 20			Healthy controls (HC) *N* = 40	BP vs. HC
Age	40.4 (10.7)			38.9 (10.0)	*t*(58) = 0.54, ns
Gender	15m, 5f			23m, 17f	*X*^2^ = 2.74, ns
Handedness R/L	17/2, 1 ambidextrous			33/5, 2 ambidextrous	*X*^2^ = 0.08, ns
Education, years	11.9 (3.7)			13.2 (3.3)	*t*(58) = 1.36, ns
Diagnosis	13 BD-I; 4 BD-II; 3 BD-NOS				
Age onset	19.2 (7.4)				
Illness duration	21.2 (10.7)				
Mood state/group	Euthymia (E)	Depression (D)	Hypomania (H)	Controls(C)	Mood/subgroup comparison^a^
Individuals	20	12	14	41	
Sessions	39	18	18	55	
YMRS	1.37 (1.67)	0.92 (1.09)	11.39 (4.13)	0.46 (0.99)	H > E = D = C
MADRS-S	3.99 (3.26)	14.19 (3.36)	2.19 (2.67)	1.17 (1.23)	D > E > H = C

Standard deviations are indicated in brackets. BD-I, BD-II, and BD-NOS refers to Bipolar Disorder type I, II and Not Otherwise Specified. Each patient completed one or several session(s) in the same mood state (according to our classification criteria). Unbalanced number of sessions were controlled for through mixed models (see Methods section).

*YMRS* Young Mania Rating Scale, *MADRS-S* Montgomery Asberg Depression Rating Scale, self-rating version.

^a^Mood and group comparisons were tested using mixed models in R.

Twenty subjects with BD were recruited from the outpatient unit “*Mood Disorder Program*” of the Geneva University Hospitals. The diagnosis of bipolar disorder was based on the DSM-IV-TR criteria and confirmed by the *Mini*-*International Neuropsychiatric Interview* [MINI; 27] administered by a trained clinician (SF, CP). Twelve patients met criteria for at least one other lifetime Axis I psychiatric disorder (see Supplementary Table S1). Eighteen patients were medicated from the first to the last session, with adaptations of drugs (9 patients) and/or doses (14 patients) by the psychiatrist during the follow-up (Supplementary Table S2).

Forty-one healthy controls were recruited. None of these participants had a history of neurological illness or Axis I psychiatric disorders as assessed by the MINI.

### Follow-up sessions

During the follow-up period (16 ± 5 months in average), the patients completed several experimental sessions with an average interval of 3 months (±2) between two successive sessions. Each session comprised a systematic psychometric assessment of mood including the clinician-rated Young Mania Rating Scale [YMRS; 28, 29], the self-rated Montgomery–Åsberg Depression Rating Scale [MADRS-S; 30, 31], and the self-rated Internal State Scale [ISS; 32]. Based on recommended cut-off scores for these scales [33, YMRS french version 29], we classified the mood state of the patients at each session into three categories labeled “hypomania” (YMRS score ≥ 6; MADRS-S score < 10), “depression” (MADRS-S score > 12, or 10 to 12 with ISS categorization into depression; YMRS < 6), and “euthymia” (MADRS-S score < 10; YMRS score < 6). Accordingly, symptoms severity was in the range of moderate to severe levels for the depression subgroup of sessions, and from “minimal symptoms” to mild mania for the hypomania subgroup of sessions.

Patients and controls completed 82 and 58 MRI sessions, respectively, among which 7 and 3 were further excluded from the analyses due to excessive motion during scanning or because subjects fell asleep. Applying the previously described psychometric criteria to our sample, we obtained data from ten patients in the three categories of mood states, from six patients in two categories, and from four patients in one category only. Table 1 (bottom part) presents an overview of the distribution of sessions included in the analyses, as well as psychometric evaluations results. Regarding medication classes, treatment regimens did not differ significantly across mood state (Supplementary Table S2). However, we further tested for changes in dosage for each single medication class in relation to our mood categories. We considered dose equivalents for antidepressants, antiepileptic benzodiazepines and atypical antipsychotics, while keeping original doses for antiepileptic mood stabilizers and testing lithium effects separately, all of which were *Z* scored (see Supplemenatry information). Higher antidepressant doses were associated with higher MADRS scores and lower YMRS scores (*t* = 2.02, *p* = 0.0482 and *t* = −2.240, *p* = 0.0292, respectively).

### Data acquisition and analysis

For each session, 150 functional images were acquired with a TR of 2.1 s while subjects were instructed to lie awake with eyes closed, and not think about anything in particular [34]. Image processing included standard procedures implemented in SPM8 (http://www.fil.ion.ucl.ac.uk/spm). Functional images were realigned, slice-time corrected, normalized to the standard Montreal Neurological Institute EPI template, and spatially smoothed with a 5 mm kernel. We discarded image volumes with frame-wise displacement above 0.5 mm and subjects (or sessions) with more than 50% of scrubbed frames [35]. No global signal regression (GSR) was carried out given the absence of agreement in the field, with some studies in favor of this procedure [36, 37], while others report spurious BOLD variance generated by GSR [38–40] or non-significant differences between dynamic FC patterns examined with and without GSR [41]. Furthermore, studies with focus on fMRI pre-processing suggest to apply further alternatives for the physiological denoising of the BOLD signal [42, 43]. Therefore, we extracted time-series of selective ROIs in the white matter and cerebrospinal fluid and we also computed the six affine motion parameters, which we then used as nuisance variables to be regressed out from the data. In addition, data were filtered between 0.01 and 0.1 Hz. For CAPs analyses, we constructed a mask based on gray matter segmentation and a probabilistic anatomical midbrain atlas including the ventral tegmental area (VTA) and the substantia nigra (SN) [44] to avoid missing reward-related brain structures relevant for BD [6].

### CAP methodology

Our objective was to extract amygdala CAPs by selecting time points when the activity of this region is high, and then averaging brain maps at the time points that shared a similar spatial distribution of activity. For this purpose, we used a combination of the left and right amygdala masks from the AAL Atlas [45] as a seed. For each fMRI session, we extracted and *Z* scored this seed BOLD time-series, and selected the 10% time points with the highest activity. We used the K-means clustering algorithm to classify the common pool of selected whole-brain volumes from all patients and controls into different clusters, for which within-cluster differences (as quantified by spatial similarity) were smaller than across-cluster differences (see [46] for more details about clustering). With a number of clusters fixed at six, the clustering showed a satisfying reproducibility level (see Supplementary information, Fig. S1), and yielded a set of CAPs representing networks with potential functional relevance and/or spatial similarity with conventional resting-state networks (see Results section). These CAPs were transformed into spatial Z-maps (normalization by the standard error), so that they quantify the degree of significance to which the CAP values deviate from zero.

For each session, we computed the fraction of the retained frames assigned to each cluster—i.e., the global occurrence rate of each CAP. We also quantified the “entry rate”, that is, the number of times that the amygdala is entering a particular state of co-activation with other brain regions, regardless of whether that state is sustained for one or several time points. We normalized the sum of entries by the total number of retained frames for the considered session. In addition, we measured mean state duration (in seconds) which features the average time for which a CAP is sustained when it appears (i.e., total number of frames assigned to the cluster, multiplied by the TR and divided by the number of entries).

Statistical analyses were performed by running linear mixed models in R. The first model assessed the effect of mood on patient’s data. In this model, the variable quantifying the CAP (e.g., occurrence rate) was modeled as a function of a fixed factor (‘mood’) with three levels: euthymia, depression, hypomania. The second model assessed both diagnostic and mood effects in the whole sample’s data using a fixed factor (‘subgroup’) with four levels (controls, BD euthymia, BD depression, BD hypomania). Subjects were included as random factor in both scenarios, enabling us to account for their unbalanced number of sessions and non-independence of repeated measures [47]. Any difference in the total number of time points assigned to a particular state of co-activation (occurrence rate metric) was further clarified by also testing for potential corresponding changes in the entry rate metric and/or in the mean duration of that state (duration metric). Age and sex effects and their interactions were also tested.

Finally, in the patients, we explored possible associations between CAP occurrences and clinical scores on the MADRS and YMRS. For that purpose, we also used linear mixed models in R by modelling CAP occurrences as a function of the clinical score (fixed factor) with subjects modeled as a random factor. As a second step, to gain further insights into any significant correlation between mania or depression global scores and CAP occurrences, we tested the same associations using entry rate and duration metrics, and using different subscores of the main clinical scales (MADRS or YMRS), which we computed based on the weights reported in Principal Components Analysis studies in larger samples of bipolar disorder patients [48, 49].

### Subsidiary analyses

Further analyses were conducted to control for potential medication effects on CAP metrics by entering original or equivalents dose when appropriate as additional regressor in the mixed models (see Supplementary information for more details).

In addition, while we assumed that both amygdalae generally display similar connectivity [50, 51] for our main analysis, some hemispheric asymmetries might nonetheless arise in relation to mood changes [52], and we therefore ran two more analyses taking either the left or right amygdala separately as a seed, to avoid missing any lateral-specific effects.

## Results

### Co-activation patterns: different network interactions of the amygdala (bilateral seed)

**Fig. 1:**
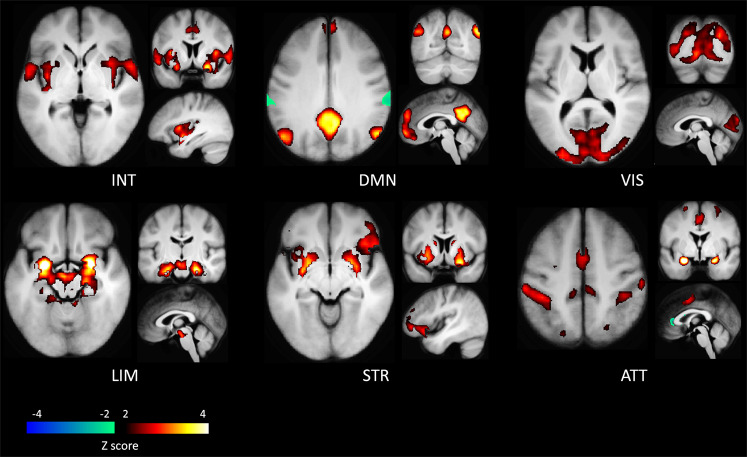
Amygdala’s co-activation patterns as obtained in the bilateral seed analysis. CAPs are named according to their description (see text): interoceptive-sensorimotor (INT), default-mode (DMN), visual (VIS), limbic (LIM), striatal (STR) and dorsal attention (ATT) CAPs. Areas in hot colors co-activate while areas in cold colors co-deactivate with the bilateral amygdala seed.

Our dynamic connectivity analyses identified six distinct CAPs, each interacting in a distinctive manner with the amygdala seed region (see Fig. 1). The first CAP (INT) involved an interoceptive-sensorimotor network and showed intense coactivation bilaterally in the middle insula, Rolandic operculum, and superior temporal gyrus, extending secondarily to the anterior middle cingulate cortex (MCC)/supplementary motor area (SMA), putamen, and finally (with *Z* score < 2) to more posterior and anterior sectors of the insula and to the central sulcus. The second CAP (DMN) overlapped with the default-mode network, comprising the posterior cingulate cortex, medial prefrontal cortex, bilateral angular gyri, and middle temporal gyri. The third CAP (VIS) represented a ‘visual network’, largely covering the occipital lobe in its inferior, middle, and superior parts, including the calcarine area and the lingual gyrus. The fourth CAP (LIM) was considered a ‘limbic network’ as it mainly included the hippocampus and parahippocampal gyrus, the VTA in midbrain, and at a lower intensity (with *Z* score < 3), the temporal pole and putamen. The fifth CAP (STR) constituted a ‘striatal network’, primarily involving the putamen and caudate, and secondarily the right inferior frontal gyrus (IFG) and MCC/medial superior frontal gyrus. Finally, the sixth CAP (ATT) overlapped with the ‘dorsal attention network’ and comprised the inferior and superior parietal gyri bilaterally, the post-central gyri, and the SMA/MCC.

### Occurrence rate, entry rate and duration of the CAPs

**Fig. 2:**
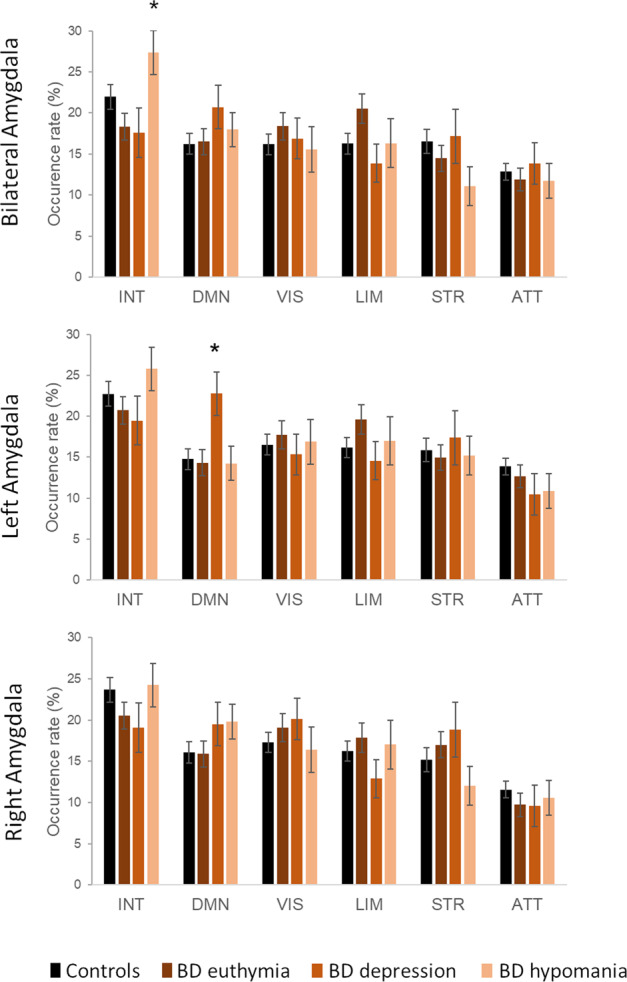
Occurrence rates of the CAPs with bilateral amygdala (top panel), left amygdala (middle panel) and right amygdala (bottom panel) used as the seed. Occurrence rates (mean and standard errors) are shown for interoceptive-sensorimotor (INT), default-mode (DMN), visual (VIS), limbic (LIM), striatal (STR) and dorsal attention (ATT) CAPs, for controls and for patients depending on mood. Starts indicate significant mood comparisons within the patients group (see results in Table 2). Error bars indicate standard errors.

After computing the temporal metrics for each CAP, we first compared the occurrence rates between each group and each mood state (Fig. 2), and then clarified any difference by testing entry rate and duration metrics. Statistically significant findings related to mood comparisons and association with clinical scores are reported in Table 2.

**Table 2:** Results of the analyses of the occurrence rate and entry rate of the CAPs for bilateral and unilateral left amygdala seeds.

SEED	Mixed model	Main effect of Mood	*β* estimates (SE)			*P value*
			*Euthymia (E)*	*Depression (D)*	*Hypomania (H)*	
BILAT.	INT-CAP					
	Occurrence rate	*F* = 4.1, *p* = 0.021	17.7 (2.4)	17.9 (3.3)	27.3 (3.2)	H > E, *p* = 0.009 H > D, *p* = 0.027
	Entry rate	*F* = 4.7, *p* = 0.012	19.0 (2.3)	17.1 (3.0)	27.1 (2.9)	H > E, *p* = 0.011 H > D, *p* = 0.008
LEFT	DMN-CAP					
	Occurrence rate	*F* = 3.2, *p* = 0.048	14.3 (2.0)	22.8 (2.9)	14.2 (2.9)	D > E, *p* = 0.020 D > H, *p* = 0.043
	Entry rate	*F* = 4.6, *p* = 0.014	14.0 (1.7)	22.7 (2.5)	13.8 (2.5)	D > E, *p* = 0.006 D > H, *p* = 0.015
SEED	Regression in the patients			β estimates (SE)	t	p
BILAT.	INT-CAP					
	Occurrence rate	YMRS	Global mania	0.73 (0.29)	2.54	0.014
		Y1	Irritable mania	2.81 (0.98)	2.87	0.006
		Y2	Elated mania^a^	2.33 (1.11)	2.11	0.039
	Entry rate	YMRS	Global mania	0.65 (0.26)	2.56	0.013
		Y1	Irritable mania	2.4 (0.87)	2.78	0.007
		Y2	Elated mania^a^	2.05 (0.98)	2.10	0.040
LEFT	DMN-CAP					
	Occurrence rate	MADRS	Global depression	0.82 (0.25)	3.29	0.002
		M1	Sadness	3.45 (1.01)	3.41	0.001
		M2	Negative thoughts	2.74 (0.74)	3.69	0.0004
		M3	Detachment	2.57 (0.81)	3.16	0.002
	Entry rate	MADRS	Global depression	0.77 (0.22)	3.56	0.0007
		M1	Sadness	3.30 (0.88)	3.77	0.0003
		M2	Negative thoughts	2.46 (0.65)	3.79	0.0003
		M3	Detachment	2.23 (0.71)	3.31	0.001
		M4^a^	Neurovegetative symptoms	2.27 (1.04)	2.18	0.032

Significant effects of mood (top panel) and significant association with the clinical variables in the patients (bottom panel) are reported.

^a^The association does not survive when antidepressant medication dose is controlled for (see Supplementary Information).

#### Bilateral amygdala’s co-activation patterns

For the ‘INT’ (interoceptive-sensorimotor) CAP, a mixed model analysis including the patients only revealed a significant main effect of mood on occurrence rate. Accordingly, this CAP occurred more often during hypomania than during euthymia and depression. Further analyses showed that the entry rate for INT was also increased during hypomania compared to euthymic and depressed states (Fig. 2). In addition, across the patients, higher occurrence rate and higher entry rate for INT were both associated with more severe mania (total YMRS score, see Table 2). To understand the relationship between INT and mania, we tested the same associations by taking subscores of this scale representing three different aspects of mania [48]. We found that the factor ‘irritable mania’ (Y1) and, to a lesser extent, ‘elated mania’, were positively associated with both the occurrence rate and the entry rate (Table 2) whereas ‘psychotic mania’ was not. By contrast, the mean duration of CAP_1_ was not affected by mood state and not related to clinical scores. Finally, comparing INT across the four different participant/mood subgroups, we find a significant effect of subgroup for the entry rate (*F* = 2.9, *p* = 0.040), mainly driven by a higher rate in hypomanic patients than controls (*ẞ* (SE) = 27.1(2.9) vs. 20.6(1.7), *p* = 0.056). The main effect of subgroup was not significant for the global occurrence rate (*F* = 2.6, *p* = 0.058).

#### Unilateral amygdala’s co-activation patterns

Our analyses based on unilateral seeds yielded similar CAPs in terms of spatial distribution and temporal dynamics, although the main effect of mood on the interoceptive CAP was statistically significant only for the bilateral seed analysis due to a probable synergic effect of the interhemispheric dynamic FC of both amygdalar regions. Importantly, we observed a specific effect of mood in the left amygdala-based CAPs analysis which was absent for the right amygdala analysis. For the left amygdala, the occurrence rate of the DMN CAP was increased in patients specifically during the depressed mood state, in comparison to euthymic and hypomanic state (see Table 2), and in comparison to controls as well (subgroup effect). Indeed, we observed during depression an increase in the entry rate of this CAP, not in the mean duration. Importantly, both occurrences and entry rate metrics were positively associated with MADRS score and subscores as well (Table 2).

We did not observe any sex or age effects interacting with the reported effects.

### Control for medication effects

In terms of medication, we identified in the patients a general effect of taking antidepressants, which was associated with a decrease in INT-CAP occurrence rates (bilateral seed) and an increase in DMN CAP occurrence rates (left seed). However, doses of medication were not collinear with, and did not interact with, mood or clinical scores. The reported associations between CAP occurrences and clinical scores were still significant even after controlling for antidepressant effect, except for the elated mania subscore (Y2) and INT CAP metrics (see Supplementary information). These findings indicate that, although medication may be associated with changes in the temporal brain dynamics of BD patients, such effects are unlikely to explain the specific alterations that we described in relation to mood changes.

## Discussion

We explored the spatiotemporal dynamics of amygdala connectivity at rest in 20 BD patients longitudinally followed-up across different clinical phases through repetitive scanning sessions. Our CAP analysis highlighted that, among the six different networks whose activity transiently co-varied with the amygdala, two were modulated across the three clinical states during the longitudinal follow-up. First, the temporal dynamics of the interoceptive-sensorimotor CAP were consistently affected by current mood, with a higher occurrence rate of this pattern during hypomania than during the other mood states. This effect appeared to be a synergic effect of both amygdalae activations leading to a significant main effect of mood on the co-activation frequency of this network with the bilateral seed only. Importantly, increased expression of this interoceptive CAP was associated with more severe manic symptoms. Second, separate analyses with unilateral seeds revealed an increased co-activation of the left amygdala with the DMN in the patients specifically during depression. Again, this latter increase was associated with the severity of depression symptoms.

INT CAP is predominantly composed of bilateral structures involved in interoceptive processes, namely the middle and posterior parts of the insula [53, 54], which receive visceral information important for homeostatic regulation. The network also comprises elements of the sensorimotor systems including co-activated regions in the lateral and inferior sectors of somatosensory and motor cortices in the Rolandic operculum, precentral gyri, and central sulcus, as well as the putamen [55], cingulate motor areas [56], and the SMA. The more frequent co-activation of the viscerosensory areas (middle and posterior insula) and the amygdala during manic phases fuels the debate on interoception disturbances in mood disorders [57–59]. According to computational perspectives, interoception can be modeled as an iterative process of comparing the brain’s expectation of sensations with those coming from the sensory world or the internal milieu [58]. In case of discrepancy, prediction error signals are computed in the viscerosensory areas and transmitted to the visceromotor (control) areas (e.g., anterior insula and anterior ACC), which can initiate an adequate adjustment [60]. Some authors argue that mood acts as a hyperprior over emotional states, i.e., mood can bias the confidence we place in our prior beliefs relative to sensory evidence [61]. In this model, mania would be associated with hyperprecise predictions, resistant to negative feedback loops (i.e., lacking adjustment to the actual sensory signal), with prediction bias toward rewarding and predictable environment [61, 62]. Given the central role of the amygdala in the predictive interoceptive circuitry [60, 63], our findings are compatible with such a prediction bias, which may entail a co-occurrence of heightened arousal signals in the amygdala and increased error signals in the viscerosensory areas. Thus, the present findings bring new arguments in favor of the conceptualization of BD as an “interoceptive psychosis” [64]. These results also converge with accumulating evidence from resting-state FC studies concerning the sensorimotor networks in BD disorders [5, 13, 65, see also 66] as well as increased connectivity observed between the amygdala and motor [e.g., SMA, 67] or sensory networks [among others, 2]. The dysbalancing of intrinsic brain activity toward sensorimotor patterns in mania is actually supported by prior investigations on the various phases of BD using different resting-state fMRI measurements [13, 68, see also 69]. In addition, a recent model on neurotransmitters— resting-state networks interactions suggests that a concomitant over-activation of the SMN and salience network (including the insula) contributes to manic symptomatology [70]. Interestingly, consistent with our interpretation above, the occurrence of INT-CAP was especially associated with the factor ‘irritable mania’ from the YMRS scale—a factor that reflects, primarily, symptoms of irritability and increased motor activity/energy, and secondarily, disruptive-aggressive behavior [48]. This could result from the heightened reactivity of motivation-related error signals mediated by dynamic amygdala-interoceptive circuits. Altogether, these findings are consistent with a form of hypersalience processing [2] and perhaps denote a stronger affective meaning of somatosensory experiences and exaggerated arousal during manic states.

The second important finding of this study highlights a special role of the left amygdala and its interaction with the DMN during depression state in our BD patients. Beyond its task-negative profile [71], this network is deemed as a key brain system underpinning internal thoughts, mind wandering, autobiographical or prospective memory, and social cognition [72, 73]. The DMN is also one of the most investigated network in bipolar and mood disorders in general [74–77]. In particular, our findings are consistent with previous suggestions of a dysbalance among intrinsic networks in favor of the DMN during depression [13, 68, 70], revealed here through its interaction with the left amygdala. Remarkably, amygdala’s coupling with the DMN (or some of its hubs) has previously been documented in resting-state studies of BD [52], and was suggested to be associated with rumination processes [e.g., 11, 78] and internalizing symptoms [e.g., 79]. The selective left-sided effects might accord with some form of internal speech or mainly verbal contents of ruminative thinking coupled with left amygdala activity. However, other works have emphasized another functional perspective on the DMN system. According to Yeshurun et al. [72], the DMN is a ‘sense-making’ network that integrates moment-by-moment incoming external information together with one’s internal pieces of information. In the same line, a recent study in healthy participants found increased DMN CAP occurrences at rest following the presentation of sad movies, by contrast with neutral movies [80], suggesting that DMN dynamics is shaped by affective experience. In this context, we might interpret the amygdala-DMN dynamic interaction as a neural mechanism integrating internal information, related to personality traits, long-term emotional memories, or mental schemata, with the processing of currently incoming inputs, contributing to the content of thoughts occurring at rest [see also 81, 82]. Accordingly, the increased frequency of DMN-amygdala co-activation during depression might be compatible with patients’ erroneous or biased interpretations of their internal or external world associated with negative thoughts and sadness.

In conclusion, we report two different results that could contribute to different clinical symptomatology during mania vs. depression mood switches. On one side, the bilateral amygdala exhibits increased transient interactions with an interoceptive-sensorimotor network during hypomania. This co-activation state appears more frequently under elevated mood states, and its association with manic symptoms seems to be driven by irritability and motor agitation. This abnormal dynamic interaction between emotion processing and interoception/sensorimotor networks might underlie the heightened arousal state of mania. On the other side, the left amygdala showed increased interaction with the DMN during depression state. Such modulation of amygdala-DMN temporal dynamics might be compatible with, for instance, an affective interpretation bias associated with depressive symptomatology and self-reflexive ruminative thinking. In our views, these findings are compatible with the three-dimensional theoretical model by Martino and Magioncalda [83], which predicts a predominance of the psychomotor and affective “units” (mainly involving SMN and SN, respectively) in the hypomanic state. Although we did not observe the predicted decrease in these units during depression, our results support that the predominance of the associative unit (mainly involving DMN), supposed to underlie thought dimension, can be observed in such a state.

Several limitations should be considered, however. Although we obtained a large number of scans in all our patients through systematic prospective follow-ups, the population is modest in terms of size and level of symptoms severity, and heterogeneous with regard to comorbidities, which altogether may restrict the generalization of our findings. In addition, we could not obtain analyzable scans in all the patients in each of the three categories of mood state as determined by our criteria. Last, our exploration of medication effects on brain dynamics should be considered with caution, as it is presently limited by the heterogeneity of patients’ medication regimens, the small number of patients taking each medication class, and potential drug interactions.

Nevertheless, this investigation supports the relevance of dynamic spatio-temporal analytical approaches to explore behavioral aspects or psychiatric symptoms that are fluctuating in nature [84]. Our findings highlight the potential of CAP analysis to unveil specific spatiotemporal mechanisms in brain systems that could be associated with depressive and manic symptomatology [85].

## Funding and disclosure

This research was supported by grants from the BRIDGE Marie-Curie COFUND action (number 267171) under the FP7 program (GR), the NARSAD Independent Investigator Grant (#22174) provided by the Brain & Behavior Research Foundation (DV), as well as the Boninchi foundation and the Foremane Chair Fund from the Geneva Academic Society (PV), the Swiss excellence Scholarship program and the Colombian Science Ministry (JG), and the Center for Biomedical Imaging (CIBM) of the Geneva-Lausanne Universities and the EPFL (MGP). The authors declare no competing interests. Open Access funding provided by Université de Genève.

## Supplementary information

Supplemental material
