# Supplementary material for: Dynamics of amygdala connectivity in bipolar disorders: a longitudinal study across mood states
Source: Neuropsychopharmacology. 2021 Jun 7;46(9):1693–701. doi: 10.1038/s41386-021-01038-x (PMC8280117; doi:10.1038/s41386-021-01038-x)
Supplement: Supplementary file 1 — Supplemental material [file 41386_2021_1038_MOESM1_ESM.docx]

Supplementary Information

**Dynamics of amygdala connectivity in bipolar disorders: A longitudinal study across mood states**

**Gwladys Rey, Thomas AW Bolton, Julian Gaviria, Camille Piguet, Maria Giulia Preti, Sophie Favre, Jean-Michel Aubry, Dimitri Van De Ville, Patrik Vuilleumier**

This material supplements but does not replace the content of the peer-reviewed paper published in Neuropsychopharmacology.

**METHODS**

*Data acquisition*

Neuroimaging data were collected using a 3T Magnetom TIM Trio scanner (Siemens, Germany) and a 32-channel head-coil. The Blood Oxygenation Level-Dependent (BOLD) contrast was evaluated using a T2*-weighted echo-planar sequence (EPI). Five hundred functional volumes of 36 axial slices each (TR/TE/flip angle = 2100 ms/30 ms/80°, FOV=192 mm, resolution=64×64, isotropic voxels of 3.2 mm^3^, distance factor 20%) were acquired in one single continuous scanning run. We collected a high-resolution T1-weighted anatomical image (TR/TI/TE/flip angle=1900 ms/900 ms/2.27 ms/9°, FOV=230 mm, resolution=256×256, slice thickness=0.9 mm, 192 sagittal slices) at the end of the first session.

*Clustering reproducibility*

To estimate an optimal number of clusters into which to subdivide the fMRI volumes with strong amygdala activation, we randomly split our full subject population (controls and BD) into two equally sized groups for a total 100 times; clustering was performed each time, separately on each half, over a range of cluster numbers from 2 to 12. Best-match pairs between each group's generated clusters were established with the Hungarian algorithm [1], and mean spatial similarity, averaged over the 100 trials, was considered as our reproducibility measure.

To assess the significance of this measure with regard to an appropriate null distribution, we created 1'000 sets of surrogate data under a stationarity assumption: white noise with the same variance as the real data was added to a subject-specific stationary activity pattern estimated from the first eigenvector of its spatial covariance matrix, and randomly scaled over time. Following temporal Z-scoring of this surrogate data, a null distribution for mean reproducibility was generated for each cluster number, from 2 to 12, as described above.

As seen in Figure S1 above, all cluster numbers larger than 4 in the assessed range of values display a mean reproducibility that exceeds the 99^th^ percentile of the associated null distribution, which means that relevant dynamic information lies in the data at all those different scales of analysis. The difference then resides in the increased subdivision of obtained co-activation patterns into smaller subnetworks. Here, we opted for a trade-off value of 6, as observed co-activation patterns matched well-known resting-state networks examined in previous functional connectivity studies.

**SUBSIDIARY ANALYSES**

*Control for medication effects*

To estimate dose equivalents for each medication class, we used chlorpromazine-equivalent dose for atypical antipsychotics (quetiapine fumarate, aripiprazole, olanzapine, risperidone, amisulpiride), clonazepam-equivalent dose for antiepileptic benzodiazepines (pregabalin, lorazepam, oxazepam), and escitalopram-equivalent for antidepressants (bupropion, venlafaxine, paroxetine)[2,3,4].

We kept original dosage for antiepileptic mood stabilizers (valproate and lamotrigine) and analyzed lithium effects as a single variable, all of which were z-scored before entering the mixed models (CAP metrics analysis) or flexible model (seed-based correlation analysis).

- Interoceptive CAP (bilateral amygdala) & antidepressants

We observed a main effect of antidepressants (AD) on INT CAP occurrences using the bilateral seed; the five patients taking antidepressants (19 sessions) had globally fewer occurrences of INT-CAP1 than the other 15 patients (56 sessions), with INT CAP occurrence rates at 11,6 ± 11,7% (with antidepressants) against 23,3 ± 13,1% (without). Considering the lack of collinearity between mood (or clinical scores) and AD dosage, and the lack of interaction between them (F=0.3, p=0.7), we applied a generalized additive mixed model which revealed a main effect of AD (F=10.4, p=0.004), in addition to a main effect of mood (F=3.6, p=0.034). In addition, there was no effect of antidepressant dosage on INT-CAP occurrences in the patients who were taking this medication (F=0.4, p=0.5). Finally, we observed that the association between YMRS score and INT-CAP occurrences (and entry rate) remained significant when AD dosage was included in the model (t=2.16, p=0.003). The association was also still significant with the mania subscore Y1, but not Y2.

- DMN CAP & antidepressants

A simple regression showed a positive association of antidepressant dosage with DMN CAP occurrences using the left seed (t=2.0, p=0.045). In the absence of collinearity and interaction between AD dosage and mood, and similarly between AD dosage and MADRS score, we applied additive models for occurrences and entry rates. In these models including both a clinical variable (mood or MADRS score) and AD dose, the association of the DMN CAP metrics and AD disappeared. The main effect of mood on occurrences disappeared (F=2.1, p=0.13) while that on entry rates remained significant (F=3.1, p=0.05). In addition, DMN CAP occurrences and entry rates were still positively associated with the MADRS score (t=2.7, p= 0.009; t=2.9, p=0.005), as well as with subscales M1, M2, M3. These findings indicate that AD medication may participate to increase DMN CAP occurrences in all the patients (whatever mood state), but does not explain increased DMN CAP occurrences in BD patients during depression specifically.

*Control for ‘seed-selectivity’ : CAPs based on primary visual cortex*

To illustrate that the effects reported with amygdala seed are specific to the selected seed, we ran another analysis with the visual cortex as a ‘control’ brain area supposedly unrelated to bipolar disorder. As seed, we selected the primary visual area V1 [5] using the Anatomy toolbox [6].

Our CAPs analyses based on V1 yielded 6 networks essentially composed of activity in the visual areas. As shown in Figure S2, some activity in conventional or known networks also showed up in addition to the visual areas, such as the DMN in CAP3, SMN in CAP4, and some little activation in areas also observed your interoceptive amydala-based CAP (superior temporal, middle insula and middle cingulate) in CAP6.

Group and mood comparisons using the same mixed models as with the amygdala showed a principal effect of subgroup (including controls) and of mood (patients only) on occurrence rates for CAP1 only (Figure S3).

**References**

1. Kuhn HW. The Hungarian method for the assignment problem. Naval Research Logistics Quarterly. 1955;2:83–97.

2. Hayasaka Y, Purgato M, Magni LR, Ogawa Y, Takeshima N, Cipriani A, et al. Dose equivalents of antidepressants: Evidence-based recommendations from randomized controlled trials. Journal of Affective Disorders. 2015;180:179–184.

3. <https://www.psychiatryinvestigation.org/journal/Table.php?xn=pi-14-647.xml&id=T1-pi-14-647&number=406&p_name=0632_406>

4. <https://clincalc.com/Benzodiazepine/>

5. Amunts K, Malikovic A, Mohlberg H, Schormann T, Zilles K. Brodmann’s areas 17 and 18 brought into stereotaxic space-where and how variable? Neuroimage. 2000;11:66–84.

6. Eickhoff SB, Stephan KE, Mohlberg H, Grefkes C, Fink GR, Amunts K, et al. A new SPM toolbox for combining probabilistic cytoarchitectonic maps and functional imaging data. NeuroImage. 2005;25:1325–1335.

Table S1. Detailed clinical description of the patients.

Twelve patients met criteria for at least one other lifetime Axis I psychiatric disorder.

| ***Lifetime presence of*** | ***Number of patients*** |
| --- | --- |
| ***- Axis 1 disorder:*** |  |
| General anxiety disorder | 4 |
| Panic disorder | 1 |
| Post-traumatic syndrome disorder | 1 |
| Obsessive-compulsive disorder | 1 |
| Social phobia | 2 |
| Attentional deficit and hyperactivity disorder | 3 |
| Substance use disorder¹ | 10 |
| ***- Other characteristics*** |  |
| Psychotic symptoms | ≥8 |
| Suicide attempt(s) | ≥5 |

¹ including alcohol (1 past, 3 current), cannabis (2 current, 1 stopped during the study), cocaine (2 past), extasy (1 past), opiate (1 past), benzodiazepine (1 past). The patients were asked to come sober the day of the experiment (sometimes short-delay appointment).

Table S2. Table of contingencies depicting the number of sessions during which patients were taking medication, as a function of mood and pharmacological class.

| Mood state  (n sessions) | Mood stabilizer | | Anti-  psychotic | | Anti-  depressant | | Benzo-  diazepine | | Psycho-  stimulant | |
| --- | --- | --- | --- | --- | --- | --- | --- | --- | --- | --- |
|  | *on* | *off* | *on* | *off* | *on* | *off* | *on* | *off* | *on* | *off* |
| Depression (n=18) | 14 | 4 | 11 | 7 | 7 | 11 | 2 | 16 | 2 | 16 |
| Euthymia (n=39) | 26 | 13 | 24 | 15 | 8 | 31 | 7 | 32 | 5 | 34 |
| Hypomania (n=18) | 14 | 4 | 12 | 6 | 4 | 14 | 6 | 12 | 3 | 15 |

Pharmacological treatment included mood stabilizers (lithium, valproate, lamotrigine), antipsychotics (quetiapine, fumarate, aripiprazole, olanzapine, risperidone, amisulpiride), antidepressants (escitalopram, bupropion, venlafaxine, paroxetine), anxiolytics (clonazepam, lorazepam, oxazepam, prégabaline), psychostimulant (methylphenidate). Treatment regimens did not differ between euthymia, depression and hypomania (Khi^2^ = 7.7, df = 18, p-value = 0.98).

Figure S2. CAPs derived from the primary visual cortex


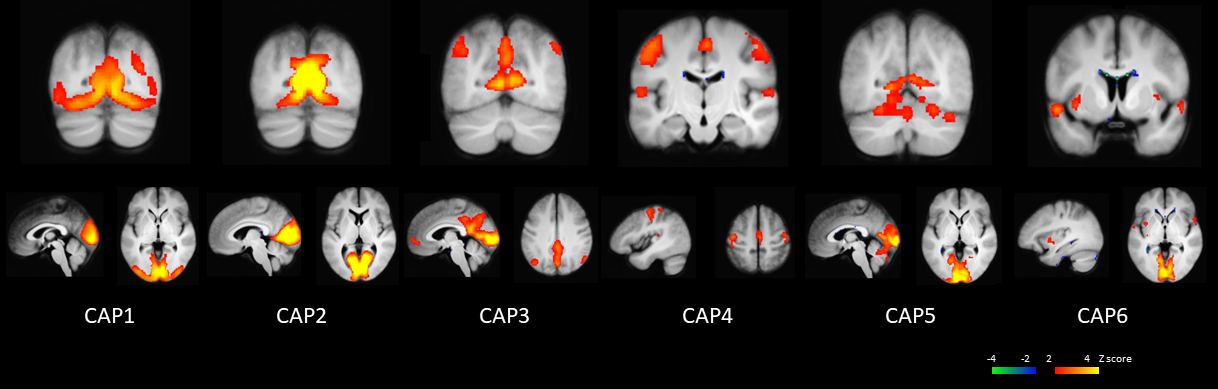


Figure S3. Occurrence rates for the six CAPs based on V1.
